# *Lyplal1* is dispensable for normal fat deposition in mice

**DOI:** 10.1242/dmm.031864

**Published:** 2017-12-01

**Authors:** Rachel A. Watson, Amy S. Gates, Elizabeth H. Wynn, Fiona E. Calvert, Amandine Girousse, Christopher J. Lelliott, Inês Barroso

**Affiliations:** 1 Human Genetics, Wellcome Trust Sanger Institute, Wellcome Genome Campus, Hinxton, Cambridge CB10 1SA, UK; 2 Department of Medical Genetics, Cambridge Institute for Medical Research, Medical Genetics, Cambridge Biomedical Campus, Hills Road, Cambridge CB2 0XY, UK; 3 Wellcome Trust-MRC Institute of Metabolic Science, University of Cambridge, Cambridge CB2 0QQ, UK; 4 Université de Toulouse, Inserm U1031, CHU Rangueil, Batiment L1, BP 84225, 31 432 Toulouse, France

**Keywords:** Adipose tissue, Genome-wide association study, *Lyplal1*, Model organism, Mouse, Obesity

## Abstract

Genome-wide association studies (GWAS) have detected association between variants in or near the *Lysophospholipase-like 1* (*LYPLAL1*) locus and metabolic traits, including central obesity, fatty liver and waist-to-hip ratio. *LYPLAL1* is also known to be upregulated in the adipose tissue of obese patients. However, the physiological role of *LYPLAL1* is not understood. To investigate the function of *Lyplal1 in vivo* we investigated the phenotype of the *Lyplal1*^tm1a(KOMP)Wtsi^ homozygous mouse. Body composition was unaltered in *Lyplal1* knockout mice as assessed by dual-energy X-ray absorptiometry (DEXA) scanning, both on normal chow and on a high-fat diet. Adipose tissue distribution between visceral and subcutaneous fat depots was unaltered, with no change in adipocyte cell size. The response to both insulin and glucose dosing was normal in *Lyplal1*^tm1a(KOMP)Wtsi^ homozygous mice, with normal fasting blood glucose concentrations. RNAseq analysis of liver, muscle and adipose tissue confirmed that *Lyplal1* expression was ablated with minimal additional changes in gene expression. These results suggest that *Lyplal1* is dispensable for normal mouse metabolic physiology and that despite having been maintained through evolution *Lyplal1* is not an essential gene, suggesting possible functional redundancy. Further studies will be required to clarify its physiological role.

## Abstract (precis)

**Summary:** We demonstrate that the *Lyplal1* gene is dispensable in mice, with important implications for interpretation of GWAS results linking *Lyplal1* to metabolism and fat distribution. This article has an associated First Person interview with the first author of the paper as part of the supplementary information.

## INTRODUCTION

Lysophospholipase-like 1 (LYPLAL1) is a protein with a poorly understood biological role, despite its evolutionary conservation (Fig. S1). The crystal structure of LYPLAL1 is similar to that of APT1 (acyl protein thioesterase 1, also known as LYPLA1), but the shape of its active site indicates that unlike APT1, which depalmitoylates Gα and Ras proteins, it cannot bind long-chain substrates. Biochemical data confirm this, also demonstrating that LYPLAL1 accepts short-chain 4-nitrophenyl esters (Bürger et al., 2012). Despite identification of a small-molecule inhibitor, the natural substrate of LYPLAL1 and its physiological role remain unknown.

Genome-wide association studies (GWAS) have identified variants close to *LYPLAL1* associated with various metabolic-related phenotypes, including waist-to-hip ratio, with a greater effect in females (Heid et al., 2010; Lindgren et al., 2009; Randall et al., 2013), subcutaneous-to-visceral white adipose tissue (scWAT/vWAT) ratio (Chu et al., 2017; Fox et al., 2012), body mass index (Benjamin et al., 2011), fasting insulin concentration, insulin resistance (Bille et al., 2011; Manning et al., 2012; Scott et al., 2012), insulin clearance (Goodarzi et al., 2013), increased fasting serum triglyceride concentration in males (Bille et al., 2011) and non-alcoholic fatty liver disease (Speliotes et al., 2011). In addition, the rs8486567 single nucleotide polymorphism (SNP) downstream of *LYPLAL1* was included in a genetic risk score associated with excess body mass loss after Roux-en-Y gastric bypass surgery (Bandstein et al., 2016).

*Lyplal1* is broadly expressed in the mouse, with higher expression in white adipose tissue (WAT), liver, skeletal muscle and kidney. *Lyplal1* expression was downregulated in a depot-specific manner in mice on a high-fat diet (HFD) (Lei et al., 2015). Likewise, *Lyplal1* expression was lower in kidney fat of Zucker diabetic fatty rats, compared with Zucker lean rats (Schmid et al., 2012), yet *LYPLAL1* expression is increased in WAT from obese patients (Steinberg et al., 2007) and in the liver in mouse metabolic disease models (Ahn et al., 2016).

Selective inhibition of *LYPLAL1* in cultured hepatocytes caused an increase in glucose production (Ahn et al., 2016), and adipocyte studies indicate that *Lyplal1* is not required for adipocyte differentiation (Lei et al., 2015).

Given previous human GWAS results, and the pathophysiological alteration in gene expression levels, we hypothesized that *LYPLAL1* is important for metabolic regulation. We characterized a *Lyplal1* knockout mouse model, aiming to elucidate the function of *Lyplal1 in vivo* and to establish whether *LYPLAL1* is a plausible causal gene at this locus.

## RESULTS

### Verification of *Lyplal1* knockout

**Fig. 1.**
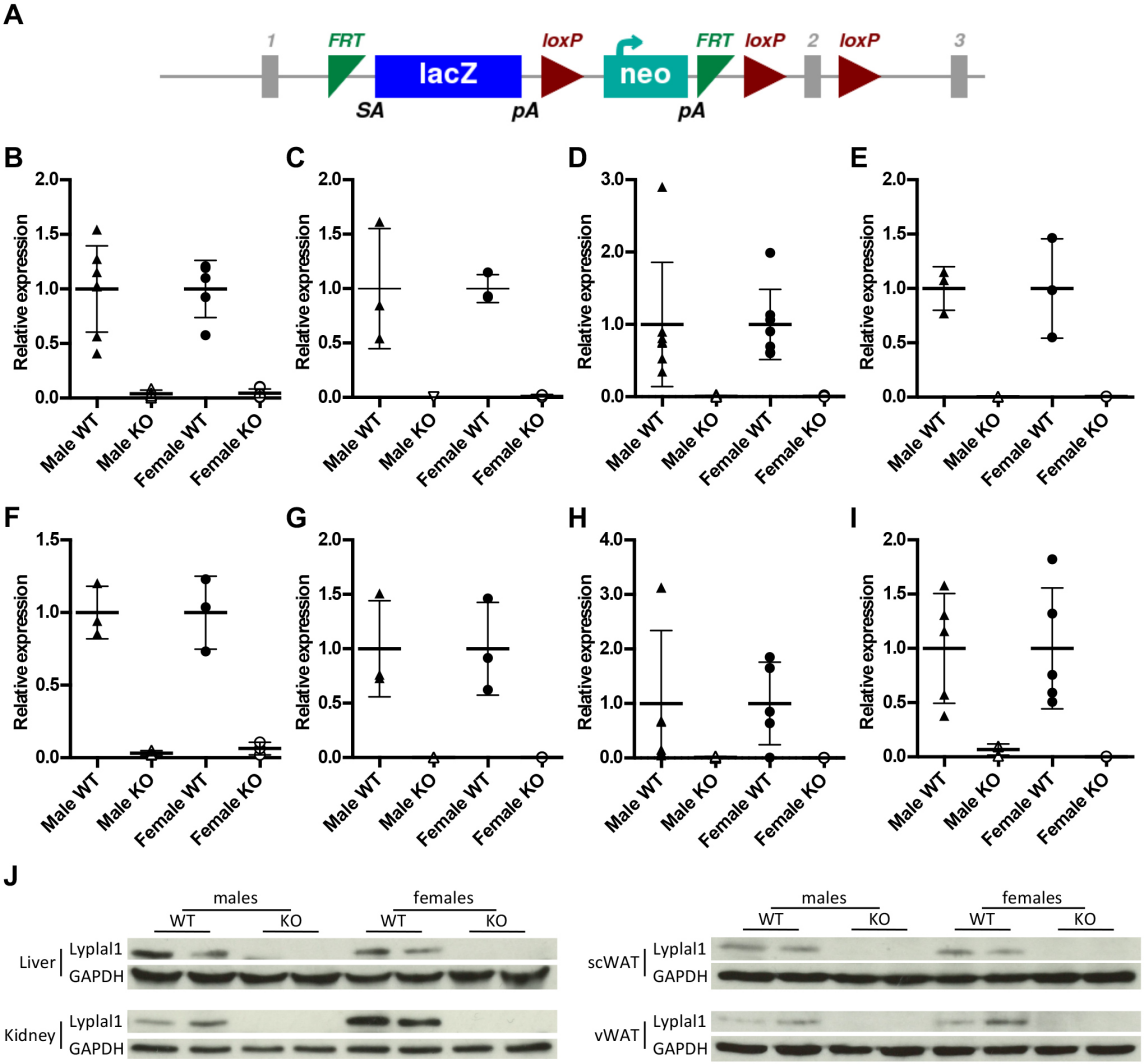
**Mice were generated with the *Lyplal1*^tm1a^ allele, resulting in knockout at both the protein and the RNA level.** (A) Diagram showing the *Lyplal1*^tm1a^ allele design (figure obtained from IMPC, www.mousephenotype.org/data/genes/MGI:2385115). RNA and protein were extracted from organs from 28-week-old mice. (B-I) qPCR analysis of *Lyplal1* mRNA levels in gastrocnemius (B), heart (C), liver (D), kidney (E), spleen (F), BAT (G), scWAT (H) and vWAT (I). Data are presented as means±s.d. Black triangles, male *Lyplal1*^+/+^; white triangles, male *Lyplal1*^tm1a/tm1a^; black circles, female *Lyplal1*^+/+^; white circles, female *Lyplal1*^tm1a/tm1a^. (J) Protein levels of Lyplal1 and GAPDH were determined by Western blot in liver, kidney, scWAT and vWAT lysates. Representative blots are shown. [(B) *n*=5 female *Lyplal1*^tm1a/tm1a^, *n*=7 other groups; (C,E-G) *n*=3 each group; (D) *n*=7 *Lyplal1*^+/+^, *n*=8 *Lyplal1*^tm1a/tm1a^; (H) *n*=8 male *Lyplal1*^+/+^, *n*=6 male *Lyplal1*^tm1a/tm1a^, *n*=5 female *Lyplal1*^+/+^, *n*=5 female *Lyplal1*^tm1a/tm1a^; (I) *n*=5 male *Lyplal1*^+/+^, *n*=3 male *Lyplal1*^tm1a/tm1a^, *n*=5 female *Lyplal1*^+/+^, *n*=6 female *Lyplal1*^tm1a/tm1a^; (J) *n*=3 samples from each sex and genotype per tissue]. KO, knockout; scWAT, subcutaneous white adipose tissue; vWAT, visceral white adipose tissue; WT, wild type.

Mice with loss of *Lyplal1* (termed *Lyplal1*^tm1a/tm1a^) were generated using the tm1a knockout first allele design as part of the International Mouse Phenotyping Consortium (IMPC) project (Fig. 1A). *Lyplal1* mRNA levels were negligible in all organs tested, with the tm1a allele resulting in >95% knockout of *Lyplal1* at the RNA level in the kidney and gastrocnemius muscle, and >99% knockout in all other tissues tested (heart, liver, spleen and adipose tissue; Fig. 1B-I). Furthermore, RNAseq confirmed loss of *Lyplal1* expression, consistent with the gene construct, with only a few detectable reads mapping to exon 1 (Fig. S2). No other exons display complete coverage in any of the *Lyplal1*^tm1a/tm1a^ samples investigated, with no more than three reads at any one base outside exon 1. The reduction in reads for exon 1 might be attributable to disruption of an unmapped regulatory element, such as an enhancer in the first exon, or a result of nonsense-mediated decay. The allele design is such that transcription is prevented beyond the lacZ in the inserted cassette. However, in the case of any skipped splicing over the cassette, the resulting transcripts would be frameshifted and subject to nonsense-mediated decay. It might also be possible that the Exon1∷LacZ transcript is detected as aberrant and degraded. *Lyplal1* was undetectable in all protein samples collected from homozygous *Lyplal1*^tm1a/tm1a^ mice (Fig. 1J; Fig. S3).

### Body composition

**Fig. 2.**
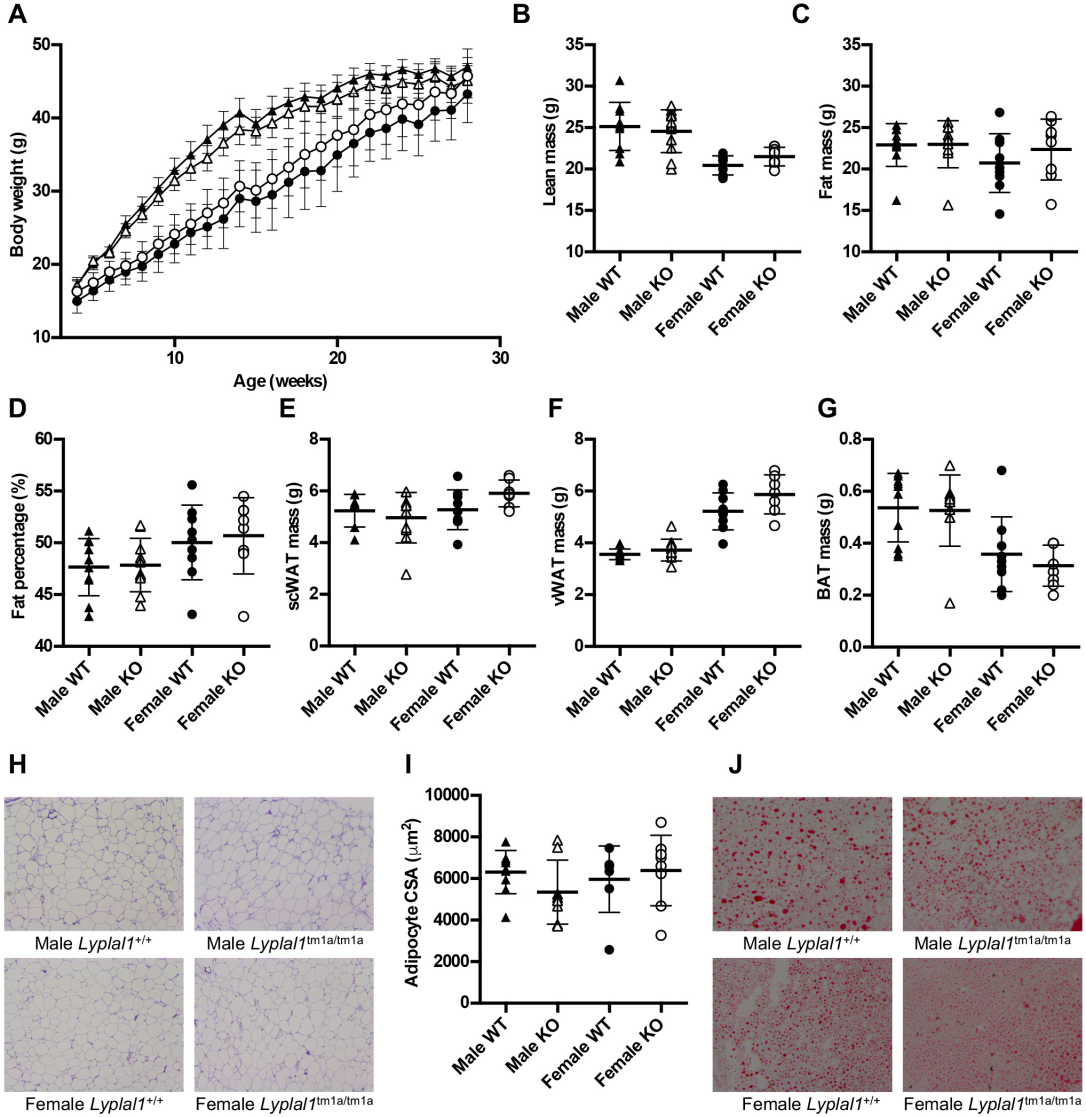
***Lyplal1* knockout does not alter body composition in *Lyplal1* knockout mice fed high-fat diet from 6 weeks of age.** (A) Body weights of mice up to 28 weeks of age were not altered by genotype. (B-D) Lean mass (B), fat mass (C) and fat percentage (D) were unaltered in 24-week-old mice, measured by DEXA. (E-G) scWAT (E), vWAT (F) and BAT (G) mass were unchanged in 28-week-old mice, weighed after dissection. (H) No qualitative change in scWAT morphology in knockout mice. Representative images are shown of scWAT sections from 28-week-old mice stained with H&E. (I) No change in adipocyte CSA was determined using ImageJ analysis of scWAT sections stained with H&E, analysed using PhenStat. (J) No change in fatty liver in knockout mice. Representative images are shown of liver sections from 28-week-old mice stained with Oil Red O and Haematoxylin. Black triangles, male *Lyplal1*^+/+^; white triangles, male *Lyplal1*^tm1a/tm1a^; black circles, female *Lyplal1*^+/+^; white circles, female *Lyplal1*^tm1a/tm1a^. Data are presented as means±s.d. Mixed model analysis was performed using PhenStat. [(B-D) *n*=10 male *Lyplal1*^+/+^, *n*=11 male *Lyplal1*^tm1a/tm1a^, *n*=9 female *Lyplal1*^+/+^, *n*=8 female *Lyplal1*^tm1a/tm1a^; (E,F) *n*=9 male *Lyplal1*^+/+^, *n*=10 male *Lyplal1*^tm1a/tm1a^, *n*=9 female *Lyplal1*^+/+^, *n*=7 female *Lyplal1*^tm1a/tm1a^; (G) *n*=10 male *Lyplal1*^+/+^, *n*=10 male *Lyplal1*^tm1a/tm1a^, *n*=9 female *Lyplal1*^+/+^, *n*=8 female *Lyplal1*^tm1a/tm1a^; (H,I) *n*=9 male *Lyplal1*^+/+^, *n*=8 male *Lyplal1*^tm1a/tm1a^, *n*=7 female *Lyplal1*^+/+^, *n*=8 female *Lyplal1*^tm1a/tm1a^]. BAT, brown adipose tissue; CSA, cross-sectional area; H&E, Haemotoxylin and Eosin; KO, knockout; scWAT, subcutaneous white adipose tissue; vWAT, visceral white adipose tissue; WT, wild type.

No obvious body weight or metabolic phenotype was observed for *Lyplal1*^tm1a/tm1a^ mice during standardized phenotyping (www.mousephenotype.org/data/genes/MGI:2385115) (White et al., 2013). To investigate further the role of *Lyplal1* on adipose tissue development, and other relevant metabolic phenotypes, mice were challenged with a HFD from 6 weeks of age. Body weight and nose-to-tailbase length were not altered in HFD-fed *Lyplal1*^tm1a/tm1a^ mice, compared with wild type (Fig. 2A; Fig. S4A,B). Lean and fat mass composition and bone mineral parameters were also unaltered in *Lyplal1*^tm1a/tm1a^ mice at 14 and 24 weeks of age (Fig. 2B-D; Fig. S4C-K). To investigate fat distribution, vWAT, scWAT and brown adipose tissue were dissected from 28-week-old mice and weighed. No change was detected in *Lyplal1*^tm1a/tm1a^ mice (Fig. 2E-G), indicating that *Lyplal1* is dispensable for adipose tissue distribution and size in mice. All other organ weights measured (liver, kidney, gastrocnemius and tibialis anterior muscles, heart and spleen) were similar to wild type in *Lyplal1*^tm1a/tm1a^ mice at 28 weeks of age (Table S1).

To determine whether adipose tissue architecture was altered, adipocyte cross-sectional area (CSA) was determined from scWAT sections (Fig. 2H-I). No changes were observed, either in average adipocyte CSA (Fig. 2I) or in the distribution of cell CSA (Fig. S5). Furthermore, liver cryosections showed no qualitative differences in *Lyplal1*^tm1a/tm1a^ mice compared with wild type (Fig. 2J), indicating that knockout of *Lyplal1* does not alter adipocyte size or the extent of fatty liver in adult mice on a HFD.

Concentrations of plasma lipids (cholesterol, high-density lipoprotein, low-density lipoprotein, non-esterified fatty acids and triglycerides) and other metabolically relevant parameters (albumin, alkaline phosphatase, alanine transaminase, amylase, aspartate aminotransferase, creatine kinase, creatinine, fructosamine and glycerol) were determined in 28-week-old mice after a 4 h fast (Table 1). No parameters were altered in *Lyplal1*^tm1a/tm1a^ mice compared with wild type, again suggesting that *Lyplal1* does not influence the regulation of these parameters.

**Table 1.**
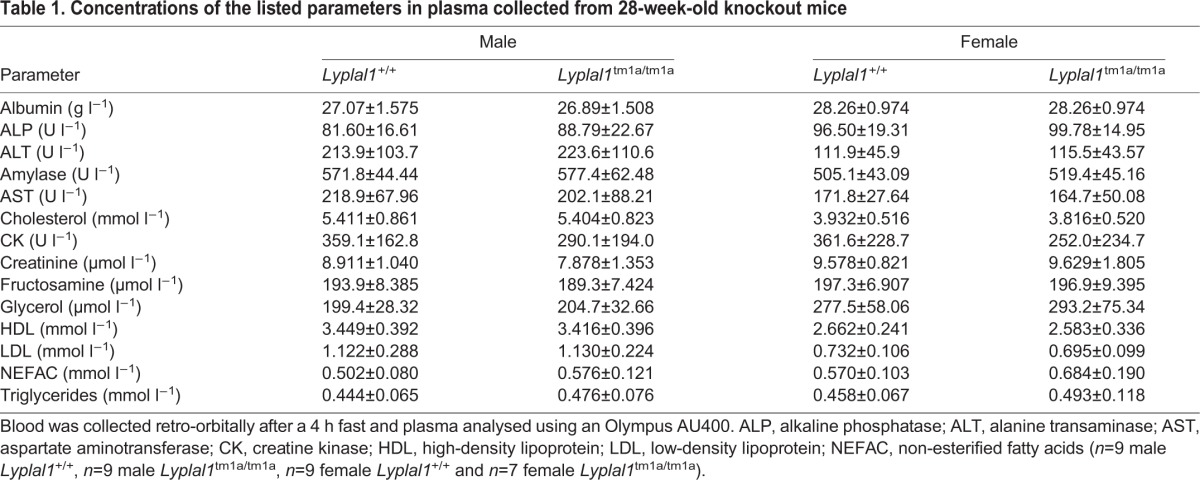
**Concentrations of the listed parameters in plasma collected from 28-week-old knockout mice**

### Glucose homeostasis

**Fig. 3.**
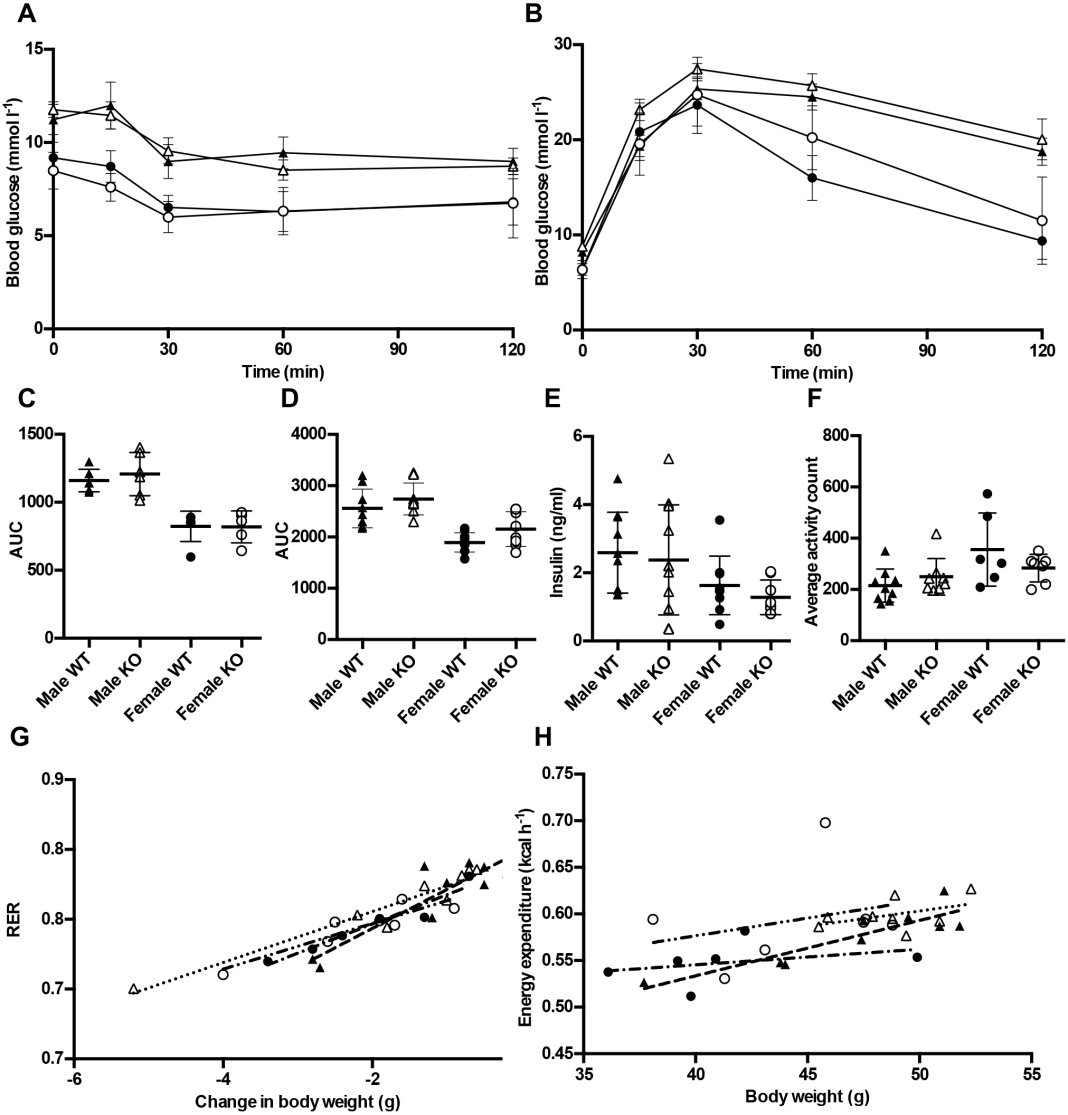
***Lyplal1* knockout does not alter glucose metabolism in mice.** (A-D) Intraperitoneal insulin tolerance test (ipITT) at 18 weeks of age (A) and intraperitoneal glucose tolerance test (ipGTT) at 22 weeks of age (B) demonstrated no alteration in glucose clearance in knockout mice. (C,D) The AUC was calculated for both ipITT (C) and ipGTT (D). AUC mixed model analysis was performed using Phenstat. (E) Plasma insulin concentrations after a 4 h fast were unaltered in 28-week-old knockout mice, determined by ELISA, mixed model analysis performed using PhenStat. (F-H) Indirect calorimetry of mice at 26 weeks of age. No differences were observed in average activity count (F), RER (G) or energy expenditure (H). Black triangles, male *Lyplal1*^+/+^; white triangles, male *Lyplal1*^tm1a/tm1a^; black circles, female *Lyplal1*^+/+^; white circles, female *Lyplal1*^tm1a/tm1a^; dashed line, male *Lyplal1*^+/+^; dotted line, male *Lyplal1*^tm1a/tm1a^; dash-dot line, female *Lyplal1*^+/+^; dash-dot-dot line, female *Lyplal1*^tm1a/tm1a^. Data are presented as means±s.d., with linear regression lines as appropriate. [(A,C) *n*=6 male *Lyplal1*^+/+^, *n*=6 male *Lyplal1*^tm1a/tm1a^, *n*=6 female *Lyplal1*^+/+^, *n*=5 female *Lyplal1*^tm1a/tm1a^; (B,D) *n*=9 male *Lyplal1*^+/+^, *n*=9 male *Lyplal1*^tm1a/tm1a^, *n*=10 female *Lyplal1*^+/+^, *n*=8 female *Lyplal1*^tm1a/tm1a^; (E) *n*=10 male *Lyplal1*^+/+^, *n*=11 male *Lyplal1*^tm1a/tm1a^, *n*=9 female *Lyplal1*^+/+^, *n*=8 female *Lyplal1*^tm1a/tm1a^; (F,G) *n*=9 male *Lyplal1*^+/+^, *n*=8 male *Lyplal1*^tm1a/tm1a^, *n*=6 female *Lyplal1*^+/+^, *n*=7 female *Lyplal1*^tm1a/tm1a^; (H) *n*=9 male *Lyplal1*^+/+^, *n*=8 male *Lyplal1*^tm1a/tm1a^, *n*=6 female *Lyplal1*^+/+^, *n*=6 female *Lyplal1*^tm1a/tm1a^]. AUC, area under the curve; KO, knockout; RER, respiratory exchange ratio; WT, wild type.

Owing to potential links with fasting insulin concentration, insulin clearance and insulin resistance, we investigated glucose homeostasis in *Lyplal1*^tm1a/tm1a^ mice. Fasting blood glucose was measured at three ages after varying durations of fast. Although concentrations differed according to sex, *Lyplal1* knockout did not alter fasting blood glucose in any conditions tested (Fig. S6). The responses to insulin and glucose doses were tested via intraperitoneal insulin and glucose tolerance tests at 18 and 22 weeks of age, respectively. *Lyplal1*^tm1a/tm1a^ mice did not show an altered response to either challenge (Fig. 3A-D). Plasma insulin concentrations in 28-week-old *Lyplal1*^tm1a/tm1a^ mice after a 4 h fast were also unaltered (Fig. 3E). Collectively, these results indicate that loss of *Lyplal1* does not profoundly alter glucose homeostasis.

### Indirect calorimetry

Indirect calorimetry was performed on 26-week-old mice for 48 h, to collect data on food intake, energy expenditure and activity levels. Activity levels and food intake during calorimetry were normal in *Lyplal1*^tm1a/tm1a^ animals (Fig. 3F; Fig. S7A,B,G,H). Although there was a small significant difference in O_2_ uptake in *Lyplal1*^tm1a/tm1a^ mice, the energy expenditure, CO_2_ output and respiratory exchange ratio remained unaltered, and traces through the time period were all qualitatively similar (Fig. 3G,H; Fig. S7C-F,J). Overall, the calorimetry did not demonstrate major changes in energy homeostasis attributable to *Lyplal1* loss.

### RNAseq

As no phenotype alterations were observed in *Lyplal1*^tm1a/tm1a^ mice, we sought to investigate whether there were compensatory changes in gene expression that might explain the apparent redundancy in *Lyplal1* function. We performed differential gene expression analysis of RNAseq data obtained from metabolically relevant tissues [liver, skeletal muscle (gastrocnemius), scWAT and vWAT]. As expected, *Lyplal1* was the top differentially expressed gene between knockout and wild type for all tissues, with additional differentially expressed genes (*P*<0.05, adjusted for multiple testing) listed in Table 2. *Lyplal1* was the only differentially regulated gene in gastrocnemius muscle, with only one additional gene differentially regulated in liver (*Nuak1*), three additional genes in scWAT and 11 additional genes in vWAT. Despite meeting the significance threshold, all these additional genes have low log-fold changes (0.2-0.75), indicating that knockout of *Lyplal1* does not cause substantial changes in the transcriptome in mice.

**Table 2.**
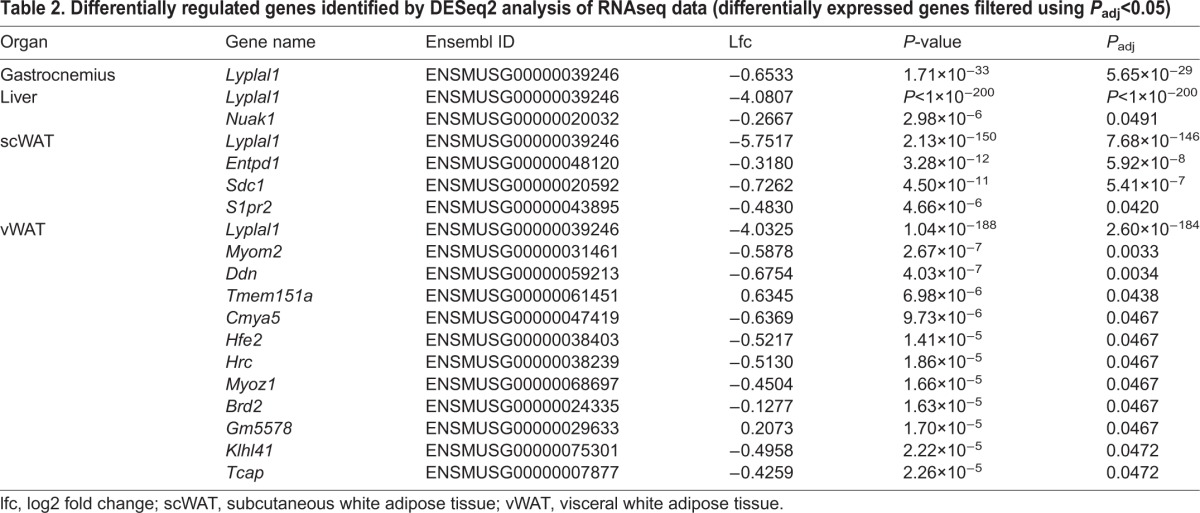
**Differentially regulated genes identified by DESeq2 analysis of RNAseq data (differentially expressed genes filtered using *P*_adj_<0.05)**

## DISCUSSION

*LYPLAL1* has been linked to many metabolic phenotypes in humans and rodents, through GWAS and expression studies (Bandstein et al., 2016; Benjamin et al., 2011; Bille et al., 2011; Chu et al., 2017; Fox et al., 2012; Goodarzi et al., 2013; Heid et al., 2010; Lei et al., 2015; Lindgren et al., 2009; Manning et al., 2012; Randall et al., 2013; Schmid et al., 2012; Scott et al., 2012; Speliotes et al., 2011; Steinberg et al., 2007). However, these results are correlative and do not demonstrate direct causality of *LYPLAL1* on associated phenotypes. We therefore aimed to investigate the *in vivo* role of *Lyplal1* in metabolic regulation and adipose tissue deposition. We studied *Lyplal1*^tm1a/tm1a^ mice obtained from the Knockout Mouse Project (KOMP) repository, with primary phenotyping obtained by the Sanger Institute Mouse Genetics Project (MGP) (White et al., 2013).

In our hands, *Lyplal1*^tm1a/tm1a^ mice did not display a detectable metabolic phenotype, even under a HFD challenge. Loss of *Lyplal1* expression was confirmed at both the RNA and the protein level, with RNAseq data showing few detectable reads mapping to exon 1, consistent with the gene construct. This suggests that the lack of obvious phenotype was not attributable to residual *Lyplal1* expression. In the absence of a detectable phenotype, there was the possibility that another gene was compensating for *Lyplal1*. However, our RNAseq data demonstrate minimal expression changes in *Lyplal1*^tm1a/tm1a^ mice, with small alterations in genes unrelated to metabolism, suggesting that *Lyplal1* is dispensable for normal fat deposition and metabolic control in mice, despite its evolutionary conservation.

Although the evidence implicating the *LYPLAL1* locus in regulation of fat distribution and metabolism was compelling, it is important to remember that the SNPs identified by GWAS are not within *LYPLAL1* itself, and the underlying causal gene or effector transcript has yet to be identified. It is therefore highly plausible that these phenotypes are regulated via other genes. This has also been well documented in the case of the identification of the causal gene around the *FTO* locus (Church et al., 2009; Claussnitzer et al., 2015; McMurray et al., 2013; Smemo et al., 2014; Stratigopoulos et al., 2014), and reinforces the caution that must be taken when interpreting GWAS results. Further studies, focused on fine-mapping the *LYPLAL1* locus and establishing a link between associated genetic variants and effects on expression and regulation of neighbouring genes, are therefore warranted to elucidate the mechanisms through which these SNPs are acting. Indeed, similar to most other GWAS loci, this variant maps to a non-coding region that might be involved in modulating gene expression rather than eliminating gene function. Therefore, modulating expression of *LYPLAL1*, and other nearby genes, in different cells, tissues and developmental stages might be required to inform the likely effector transcript at this locus and fully test whether changes in *Lyplal1* expression might contribute to the phenotype.

In conclusion, *Lyplal1* is dispensable for normal adipose and metabolic regulation in these mice for all parameters and conditions tested here. Although there are differences between mice and humans, and between mouse strains, this has important implications for the interpretation of GWAS results linking SNPs close to *LYPLAL1* to metabolic phenotypes and highlights the challenges in establishing causality based on GWAS results.

## MATERIALS AND METHODS

### Animal studies

All experiments were carried out in accordance with UK Home Office regulations, UK Animals (Scientific Procedures) Act 1986, and with approval from the Wellcome Trust Sanger Institute's Animal Welfare Committee. All mice were maintained in specific pathogen-free facilities in individually ventilated cages at standard temperature (19-23°C) and humidity (55±10%), on a 12 h dark, 12 h light (07.30-19.30 h) cycle. Sperm from C57BL6/N mice carrying the targeted, non-conditional allele *Lyplal1*^tm1a(KOMP)Wtsi^ were obtained from the KOMP repository (http://www.komp.org/pdf.php?projectID=23342; see allele design in Fig. 1A) (Skarnes et al., 2011). Following confirmation of germ-line transmission, mice derived from heterozygous intercrosses were genotyped for the *Lyplal1*^tm1a^ allele by PCR carried out as previously described (Ryder et al., 2013).

Mice underwent standardized phenotyping using a modified version of the Sanger Mouse Genetics Project pipeline detailed previously (White et al., 2013), using breeder's chow (LabDiets 5021, 21% kcal as fat; LabDiet, London, UK) instead of a HFD. *Lyplal1* data from the MGP pipeline and others is available online: http://www.mousephenotype.org/data/genes/MGI:2385115.

HFD studies using Research diets D12451 (45% kcal as fat; Research Diets, New Brunswick, NJ, USA) were performed in three batches, with 11 males and 10 females of each genotype switched from breeder's chow to HFD at 6 weeks of age. Genotypes and sexes were mixed throughout the batches.

Body composition was measured under anaesthesia with ketamine (Ketaset^®^; Fort Dodge Animal Health, Overland Park, KS, USA) and xylazine (Rompun^®^; Bayer Animal Health, Leverkusen, Germany) using a PIXImus densitometer (GE Lunar, Madison, WI, USA). Nose-to-tailbase length was measured using a ruler with 1 mm graduations prior to dual-energy X-ray absorptiometry (DEXA). This was performed at 12 (100 mg kg^−1^ ketamine and 10 mg kg^−1^ xylazine) and 22 weeks of age (90 mg kg^−1^ ketamine and 9 mg kg^−1^ xylazine). Quality control was performed using a calibrated phantom before imaging. Anaesthesia was reversed by intraperitoneal injection of atipamezole (1 mg kg^−1^ Antisedan; Orion Pharma, Espoo, Finland).

An intraperitoneal insulin tolerance test was performed on 16-week-old mice after a 6 h fast (08.00-14.00 h), using 0.6 U kg^−1^ Actrapid insulin (Novo Nordisk, Bagsvaerd, Denmark). An intraperitoneal glucose tolerance test was performed on 20-week-old mice after an overnight fast (from 17.00 h, typically 16 h duration), using 2 g kg^−1^ glucose. Mice were individually housed, and ∼0.5 mm of the tail tip was removed with a scalpel blade and a fasting blood sample directly taken (Accu-chek Aviva; Roche, Indianapolis, IN, USA). After intraperitoneal injection, further blood samples were taken at 15, 30, 60 and 120 min post-injection. Area under the curve (AUC) was calculated using GraphPad Prism.

Mice were individually housed for 48 h in indirect calorimetry cages (LabMaster system; TSE-systems, Bad Homburg, Germany) at 24 weeks of age. A final blood glucose reading was collected from 26-week-old mice after a 4 h fast, followed by anaesthesia (100 mg kg^−1^ ketamine and 10 mg kg^−1^ xylazine) and culling, with retro-orbital blood and multiple tissue samples collected.

### *Ex vivo* analysis

Heparinized whole-blood samples were centrifuged at 5000 ***g*** for 10 min at 4°C, and the separated plasma was analysed using an Olympus AU400 (Olympus, Tokyo, Japan). Insulin concentrations were determined by ELISA, according to the manufacturer's instructions (Millipore, Billerica, MA, USA).

Protein extracts were generated from frozen tissue samples by homogenization in tissue protein extraction reagent (TPER) buffer supplemented with Halt protease and phosphatase inhibitor cocktail (both from Thermo Scientific, Rockfold, IL, USA), using an Omni TH Tissue homogenizer (Omni International, NW Kennesaw, GA, USA). Western blotting of tissue protein extracts was performed using standard protocols, using the blocking solutions (in TBS with 0.1% Tween 20) and the following antibodies: rabbit anti-Lyplal1 (Proteintech Group, Rosemont, IL, USA; 1:1000), mouse anti-GAPDH (Abcam, Cambridge, UK; 1:5000), goat anti-rabbit (Bio-Rad, Hercules, CA, USA; 1:10,000) or goat anti-mouse (Bio-Rad; 1:10,000). The blocking solutions were 3% [w/v] milk (for rabbit anti-Lyplal1 and goat anti-rabbit) or 3% [w/v] BSA (for mouse anti-GAPDH and goat anti-mouse). The blots were visualized using Amersham ECL reagents (GE Healthcare, Chicago, IL, USA) and developed using a Xograph Imaging Systems Compact X4.

Paraffin sections (5 µm thickness) of scWAT were stained with Haematoxylin and Eosin (H&E) using a Leica ST5020 Multistainer machine and a Leica CV5030 Cover Slipper (Leica, Wetzlar, Germany). Cryosections (10 µm thick) of liver were stained with H&E or Oil Red O and Haematoxylin by conventional methods. Images were collected using a Leica stereomicroscope and a Hamamatsu slide scanner (Hamamatsu, Japan). Adipocyte CSA analysis was calculated from one section of 1.8 mm ×1.34 mm per mouse using ImageJ software (NIH).

### RNA extraction and qPCR

RNA was extracted from snap-frozen mouse tissue using the Qiagen RNeasy plus Universal kit according to the manufacturer's instructions (Qiagen, Hilden, Germany). RNA concentration and purity were evaluated by NanoDrop (Thermo Scientific, Wilmington, DE, USA). RNA integrity was further assessed using an Agilent Bioanalyzer (Agilent Technologies, Santa Clara, CA, USA). cDNA synthesis was performed using between 500 ng and 1 µg RNA, random primers and Superscript II reverse transcriptase (Life Technologies, Carlsbad, CA, USA). qPCR was performed using Sybr Green (Applied Biosystems, Foster City, CA, USA) and run on an AB7500 qPCR machine (Applied Biosystems). The following primers were used: for *18S*, forward 5′-GTAACCCGTTGAACCCCATT-3′ and reverse 5′-CCATCCAATCGGTAGTAGCG-3′; *Gapdh*, forward 5′-TGGTTCACACCCATCACAAACA-3′ and reverse 5′-GGTGAAGGTCGGTGTGAACGG-3′; for *Lyplal1*, forward 5′-CACGGCTCAGGTCACTCTGG-3′ and reverse 5′-AGGGGGCCGTTGGATAAATG-3′; for *Rpl32*, forward 5′-GGCCAGATCCTGATGCCCAAC-3′ and reverse 5′-CAGCTGTGCTGCTCTTTCTAC-3′. Relative expression for *Lyplal1* was calculated using the ΔΔ*Ct* method, relative to the cubic mean of three reference genes (Livak and Schmittgen, 2001).

### RNAseq

RNAseq was performed on five samples per sex and genotype from gastrocnemius, liver, scWAT and vWAT. Libraries were prepared using the Illumina TruSeq Stranded mRNA Library Preparation Kit with 10 PCR cycles (Illumina, San Diego, CA, USA). Pools of 20 samples (one pool per organ) were run on three lanes each with 75-bp paired end runs on an Illumina Hiseq 2000 with v4 chemistry, with each pool run on three lanes. Reads were aligned to the NCBI m38 version of the mouse genome, data from multiple lanes combined using Samtools v1.3, mapped reads counted using Feature Counts with duplicates retained and differential expression analysed using DESeq2 (Love et al., 2014). Outliers that did not cluster with the appropriate tissue on the principal components analysis plot were removed. The data were visualized using Integrative Genomics Viewer to confirm the location of any remaining reads in knockout samples (Robinson et al., 2011). RNAseq data are available through the European Nucleotide Archive (ENA), study number PRJEB14194 (https://www.ebi.ac.uk/ena/data/view/PRJEB14194).

### Statistical analysis

Unless otherwise stated, statistical analysis was performed as described by Karp et al. (2012), using PhenStat version 2.3.2, using the mixed model framework (Kurbatova et al., 2015). Multiple testing was managed by controlling the family-wise error rate to 5% using the Holm method (Holm, 1979).
(1)
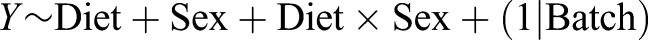
For indirect calorimetry, data analysis was performed in R, using linear model analysis, and correcting for body weight when assessing energy expenditure, O_2_ uptake and CO_2_ output, and correcting for change in body weight when assessing food intake and respiratory exchange ratio. Interaction terms were checked and were not significant; therefore, these were excluded from the model.
(2)
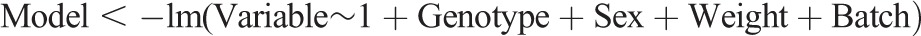


## Supplementary Material

Supplementary information

First Person interview
