# Supplementary material for: Lyplal1 is dispensable for normal fat deposition in mice
Source: Dis Model Mech. 2017 Dec 1;10(12):1481–8. doi: 10.1242/dmm.031864 (PMC5769613; doi:10.1242/dmm.031864)
Supplement: Supplementary information [file dmm-10-031864-s1.pdf]

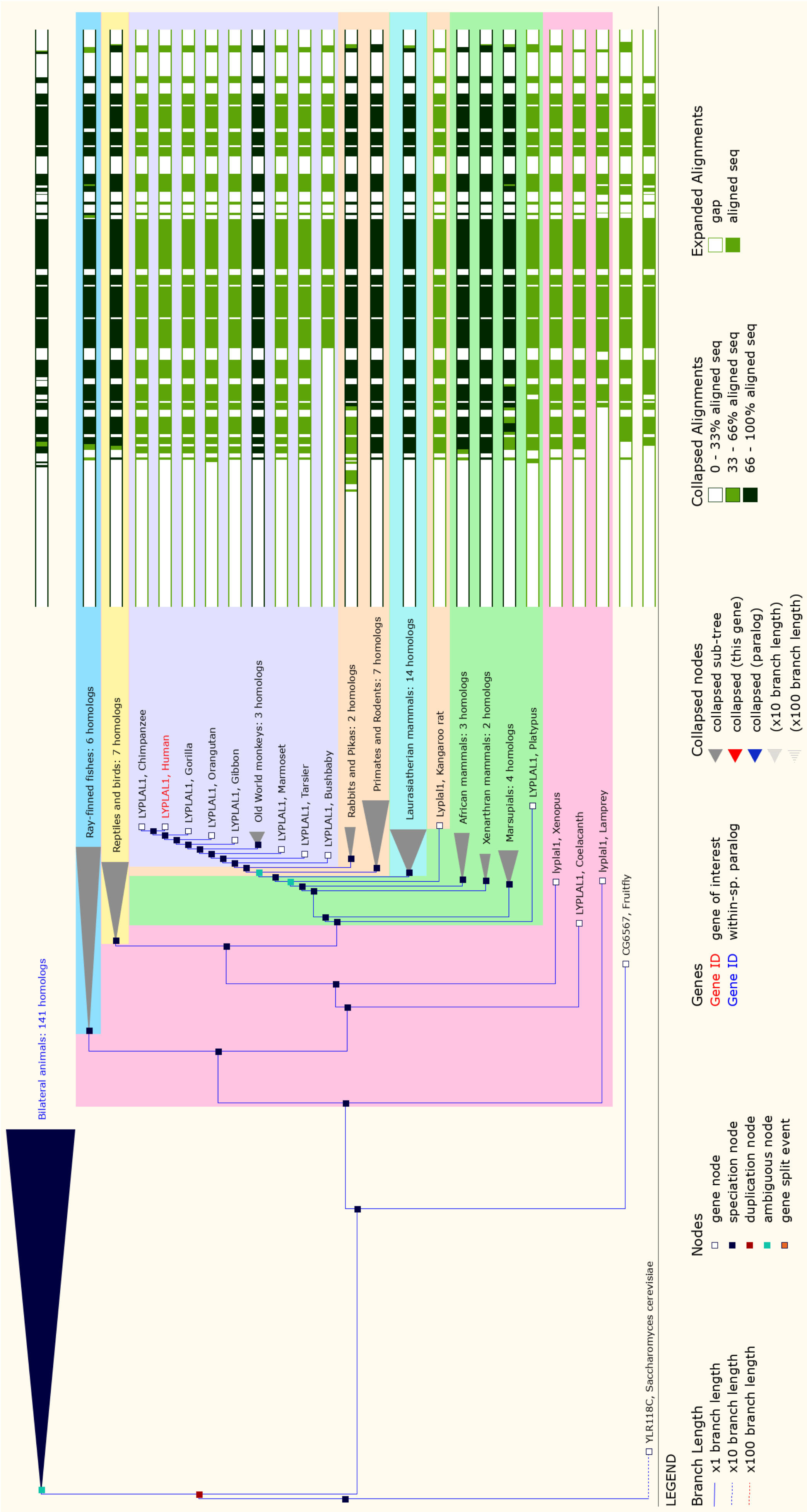

Figure S1 – Figure demonstrates the conservation of *Lyplal1* across many species. Taken from Ensembl (www.ensembl.org).

Disease Models & Mechanisms • Supplementary information

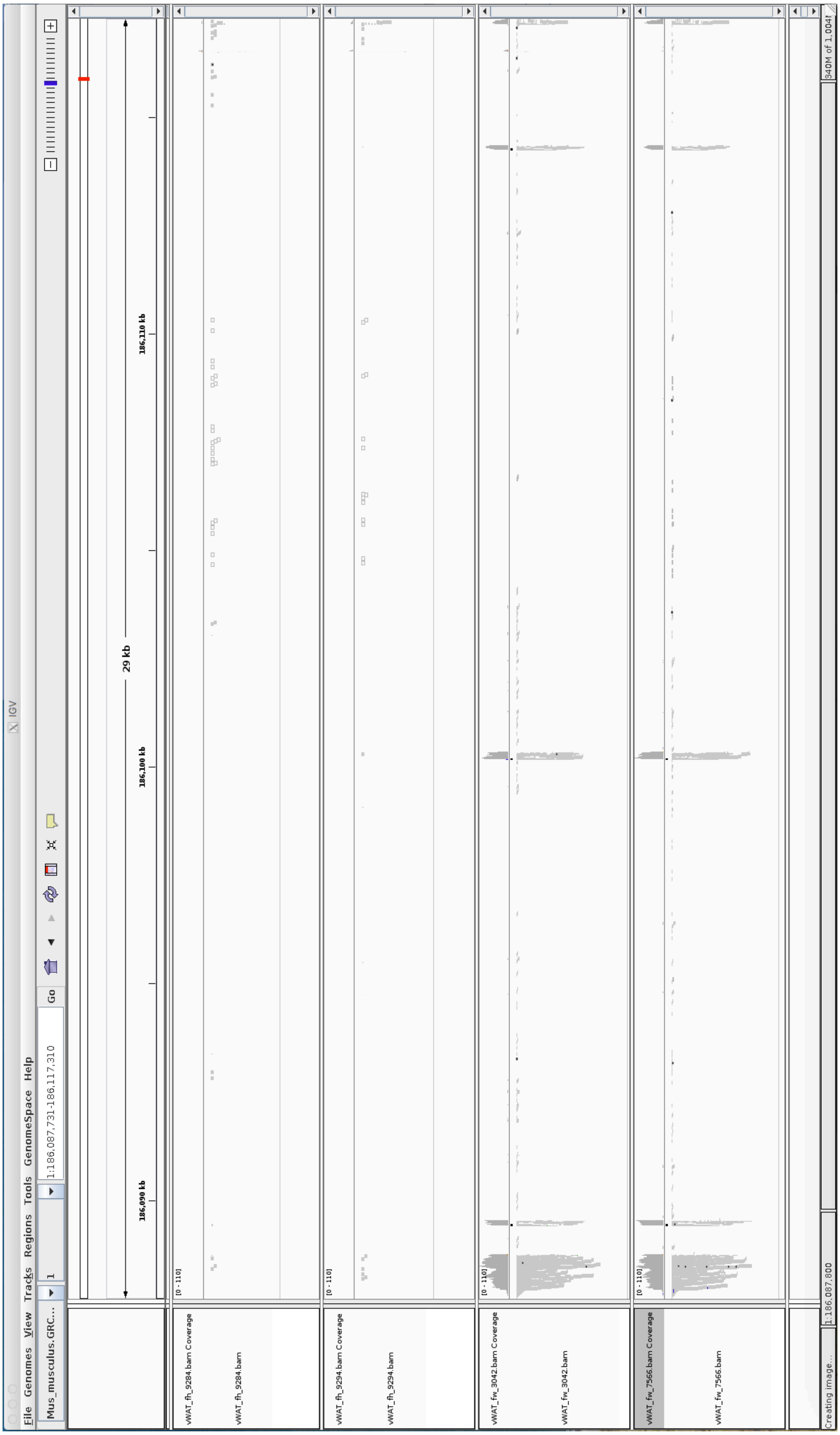

Figure S2 – RNAseq results demonstrate that there are only a few detectable reads in exon 1 of *Lyplal1*. Representative vWAT samples shown, upper two panels show wildtype samples and lower two panels show *Lyplal1*$^{tm1a/tm1a}$ samples. Figure was generated using IGV.

Disease Models & Mechanisms • Supplementary information

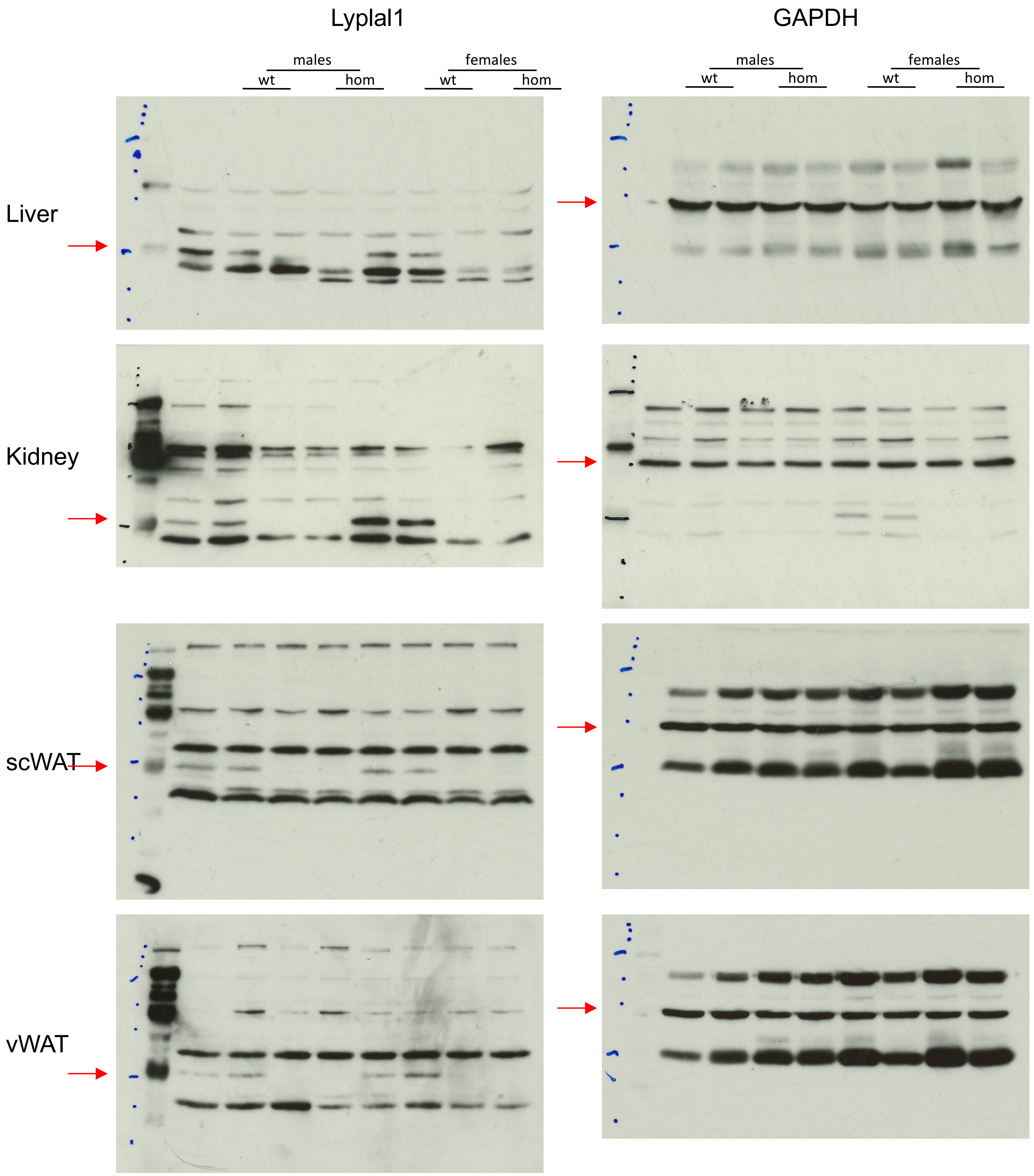

Figure S3 – Lyplal1 is knocked out at the protein level. Shown are the original Western blots. Blue marks on the left hand side correspond to Bio-Rad Dual Color Precision Plus Protein markers (250, 150, 100, 75, 50, 37, 25, 20, 15 kDa). Red arrows indicate the band for Lyplal1/GAPDH.

Disease Models & Mechanisms • Supplementary information

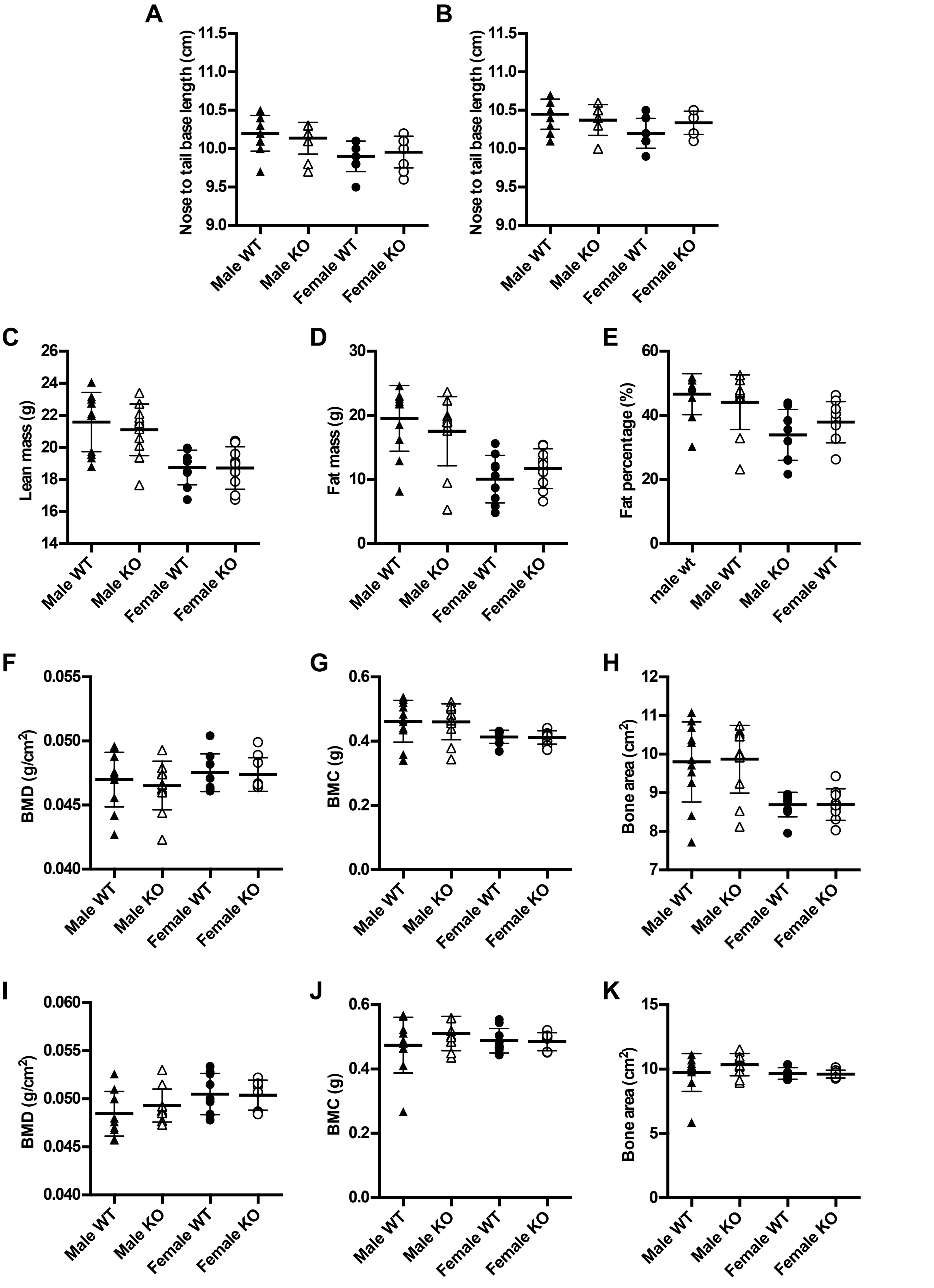

Figure S4 – *Lyplal1* knockout does not alter body composition in *Lyplal1* knockout mice fed high fat diet from 6 weeks of age.

A & B: Nose to tail base length was unaltered in 14-week-old (A) and 24-week-old (B) knockout mice.

C-H: Lean mass (C), fat mass (D) and fat percentage (E), bone mineral density (BMD, F), bone mineral content (BMC, G) and bone area (H) were unaltered in 14-week-old knockout mice, measured by DEXA.

I-J: BMD (I), BMC (J) and bone area (K) were unaltered in 24 week old knockout mice, measured by DEXA.

Black triangles = male *Lyplal1*$^{+/+}$, white triangles = male *Lyplal1*$^{tm1a/tm1a}$, black circles = female *Lyplal1*$^{+/+}$, white circles = female *Lyplal1*$^{tm1a/tm1a}$. Data are presented as means ± s.d. (n=11 males, 9 females per genotype A-H, n=10 males per genotype, n=9 female *Lyplal1*$^{+/+}$, n=8 female *Lyplal1*$^{tm1a/tm1a}$ I-K).

Disease Models & Mechanisms • Supplementary information

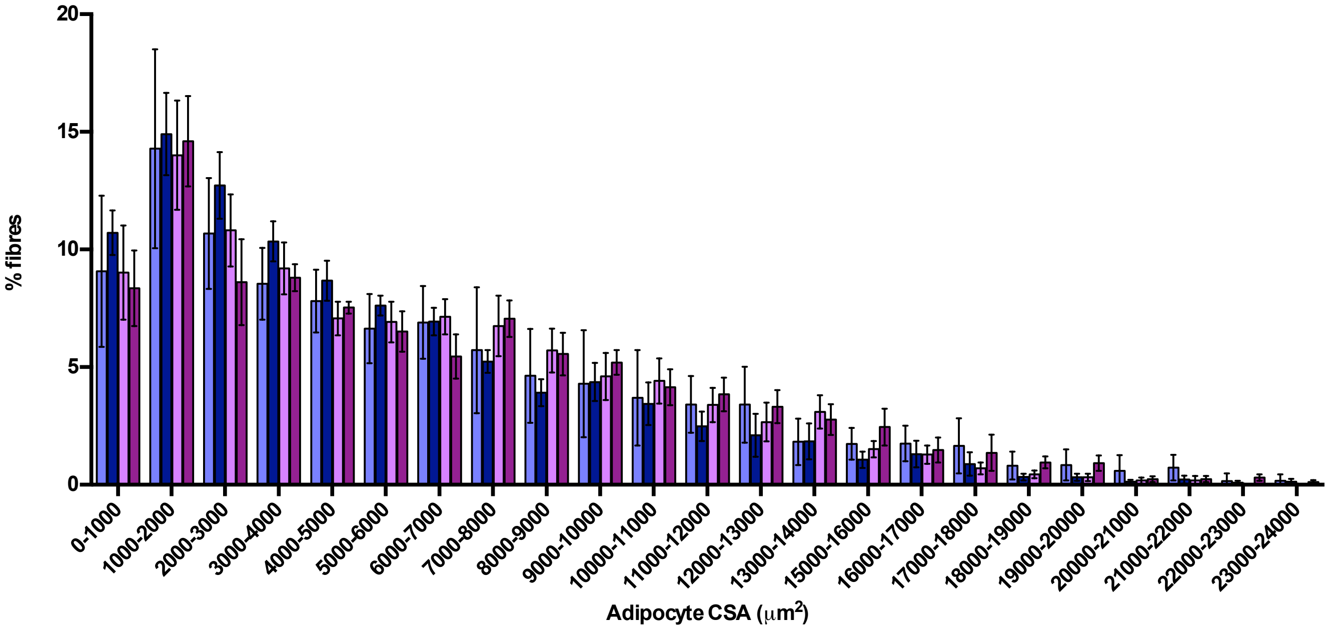

Figure S5 – *Lyplal1* knockout does not alter distribution of adipocyte CSA.
Relative adipocyte cross sectional area (CSA) was determined using ImageJ analysis of scWAT sections stained with H&E, and grouped into bins of 1000 μm^2. Light blue = male *Lyplal1*$^{+/+}$, dark blue = male *Lyplal1*$^{tm1a/tm1a}$, light purple = female *Lyplal1*$^{+/+}$, dark purple = female *Lyplal1*$^{tm1a/tm1a}$. Data are presented as means ± s.d. (n=9 male *Lyplal1*$^{+/+}$, n=8 male *Lyplal1*$^{tm1a/tm1a}$, n=7 female *Lyplal1*$^{+/+}$, n=8 female *Lyplal1*$^{tm1a/tm1a}$).

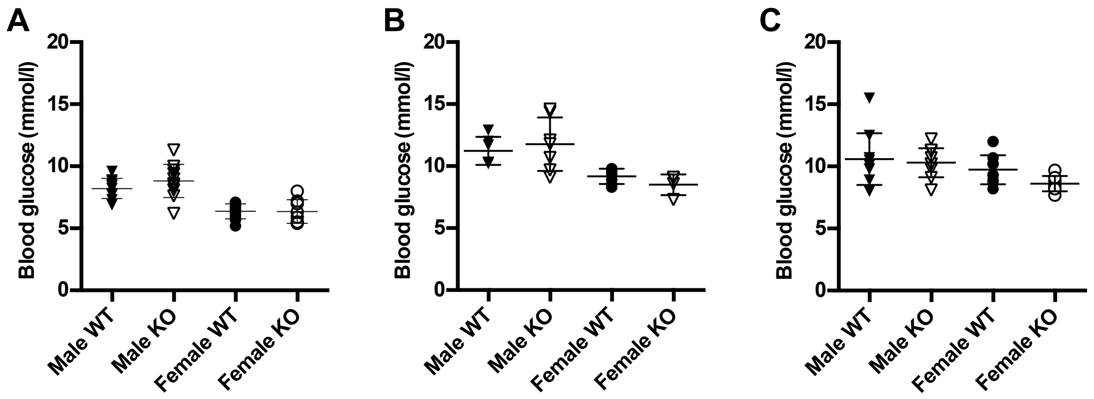

Figure S6 – Fasting blood glucose was unaltered in *Lyplal1* knockout mice after different length fasts and at different ages.

a: 22-week-old mice were fasted for 16 h overnight before blood glucose measurement. b: 18-week-old mice were fasted for 6 h before blood glucose measurement. c: 28-week-old mice were fasted for 4 h before blood glucose measurement.

Black triangles = male *Lyplal1*$^{+/+}$, white triangles = male *Lyplal1*$^{tm1a/tm1a}$, black circles = female *Lyplal1*$^{+/+}$, white circles = female *Lyplal1*$^{tm1a/tm1a}$. Data are presented as means ± s.d., mixed model analysis performed using PhenStat (A: n=6 male *Lyplal1*$^{+/+}$, n=6 male *Lyplal1*$^{tm1a/tm1a}$, n=6 female *Lyplal1*$^{+/+}$, n=5 female *Lyplal1*$^{tm1a/tm1a}$; B: n=10 male *Lyplal1*$^{+/+}$, n=11 male *Lyplal1*$^{tm1a/tm1a}$, n=10 female *Lyplal1*$^{+/+}$, n=8 female *Lyplal1*$^{tm1a/tm1a}$; C: n=10 male *Lyplal1*$^{+/+}$, n=10 male *Lyplal1*$^{tm1a/tm1a}$, n=9 female *Lyplal1*$^{+/+}$, n=8 female *Lyplal1*$^{tm1a/tm1a}$).

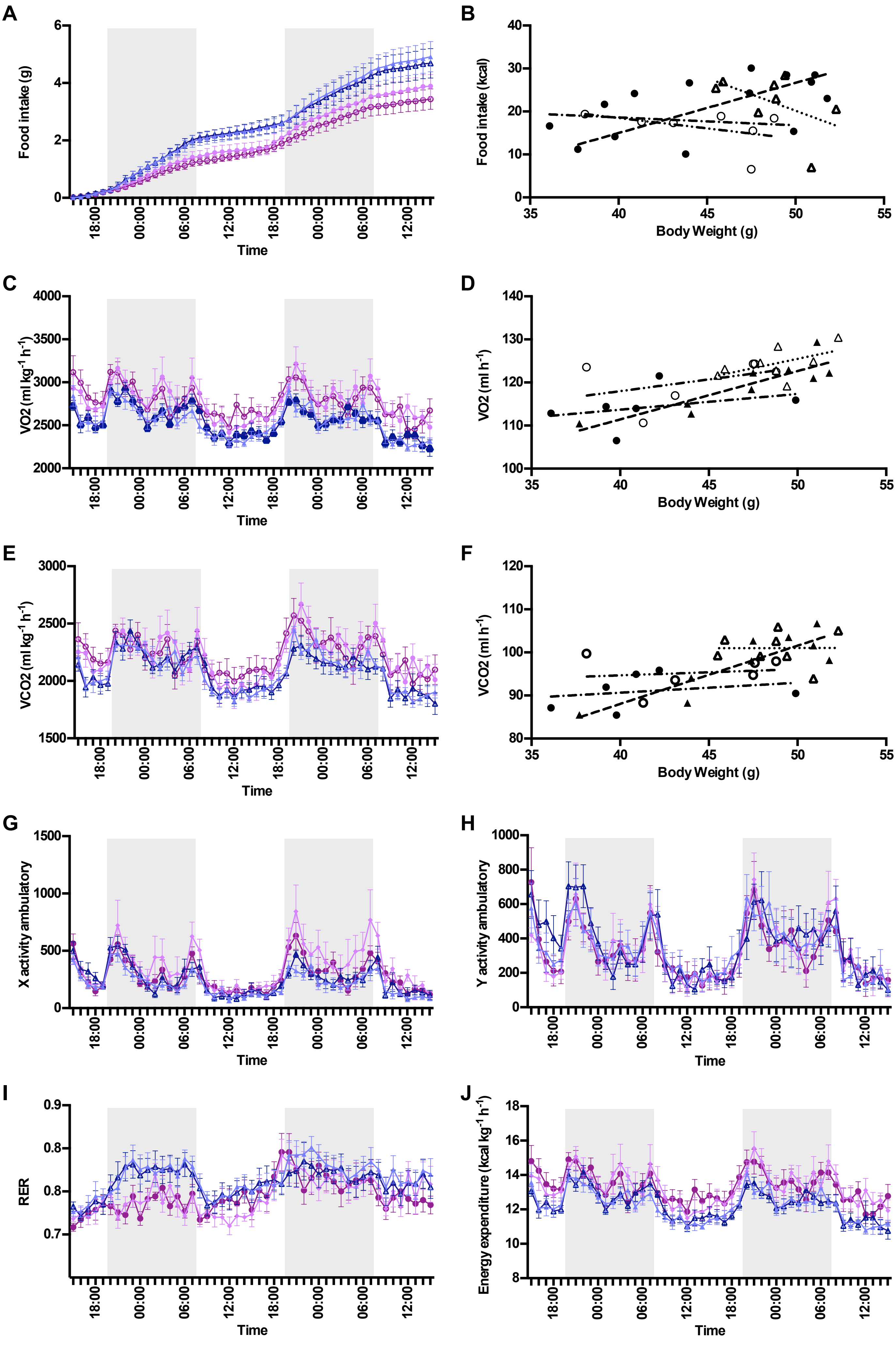

Disease Models & Mechanisms • Supplementary information

Figure S7 – *Lyplal1* knockout does not cause large changes during 48 h indirect calorimetry at 26 weeks of age. Shaded areas show the periods of dark (1930-0730).
A & B: Food intake was unaltered by genotype or sex. C & D: There was a significant effect of genotype on VO2 (p=0.038 for genotype, linear model). E & F: VCO2 was unaltered by genotype or sex. G-J: Raw data graphs for X activity, Y activity, RER and energy expenditure.
Black or light blue triangles = male *Lyplal1*$^{+/+}$, white or dark blue triangles = male *Lyplal1*$^{tm1a/tm1a}$, black or light purple circles = female *Lyplal1*$^{+/+}$, white or dark purple circles = female *Lyplal1*$^{tm1a/tm1a}$. Data are presented as means ± s.d. (A-B: n=9 male *Lyplal1*$^{+/+}$, n=8 male *Lyplal1*$^{tm1a/tm1a}$, n=6 female *Lyplal1*$^{+/+}$, n=5 female *Lyplal1*$^{tm1a/tm1a}$; C-F,J: n=9 male *Lyplal1*$^{+/+}$, n=8 male *Lyplal1*$^{tm1a/tm1a}$, n=6 female *Lyplal1*$^{+/+}$, n=6 female *Lyplal1*$^{tm1a/tm1a}$; G-I: n=9 male *Lyplal1*$^{+/+}$, n=8 male *Lyplal1*$^{tm1a/tm1a}$, n=6 female *Lyplal1*$^{+/+}$, n=7 female *Lyplal1*$^{tm1a/tm1a}$).

Table S1. No changes in mass of the listed organs in 28-week-old knockout mice. Mixed model analysis performed using PhenStat (n=10 male *Lyplal1*$^{+/+}$, n=10 male *Lyplal1*$^{tm1a/tm1a}$, n=9 female *Lyplal1*$^{+/+}$, n=8 female *Lyplal1*$^{tm1a/tm1a}$).

Organ weight (g)	Male		Female	
	Lyplal1$^{+/+}$	Lyplal1$^{tm1a/tm1a}$	Lyplal1$^{+/+}$	Lyplal1$^{tm1a/tm1a}$
Gastrocnemius	0.151 ± 0.010	0.151 ± 0.009	0.126 ± 0.006	0.141 ± 0.015
Tibialis anterior	0.076 ± 0.012	0.076 ± 0.013	0.059 ± 0.009	0.059 ± 0.009
Heart	0.157 ± 0.013	0.156 ± 0.020	0.128 ± 0.019	0.140 ± 0.012
Kidney	0.209 ± 0.021	0.209 ± 0.016	0.159 ± 0.014	0.158 ± 0.014
Liver	2.330 ± 0.706	2.405 ± 0.660	1.504 ± 0.262	1.529 ± 0.244
Spleen	0.098 ± 0.023	0.099 ± 0.016	0.104 ± 0.017	0.106 ± 0.017

Disease Models & Mechanisms • Supplementary information
