# Supplementary material for: Lyplal1 is dispensable for normal fat deposition in mice
Source: Dis Model Mech. 2017 Dec 1;10(12):1481–8. doi: 10.1242/dmm.031864 (PMC5769613; doi:10.1242/dmm.031864)
Supplement: First Person interview [file dmm-10-031864-s2.pdf]

© 2017. Published by The Company of Biologists Ltd | Disease Models & Mechanisms (2017): doi:10.1242/dmm.031864: Supplementary information

FIRST PERSON

# First person – Rachel Watson

First Person is a series of interviews with the first authors of a selection of papers published in Disease Models & Mechanisms, helping early-career researchers promote themselves alongside their papers. Rachel Watson is first author on '*Lyplal1* is dispensable for normal fat deposition in mice', published in DMM. Rachel is a postdoctoral fellow in the lab of Inês Barroso at the Wellcome Trust Sanger Institute, Cambridge, UK, investigating modelling of metabolic diseases in mice, with a focus on generating functional data for candidate genes identified through sequencing studies.

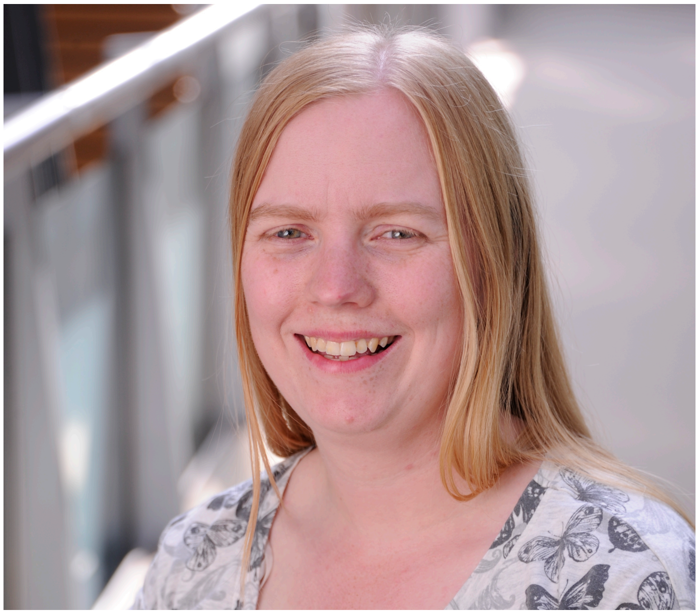

Rachel Watson

### How would you explain the main findings of your paper to non-scientific family and friends?

Studies have been performed to identify factors which increase risk of certain diseases such as obesity, diabetes and other metabolic problems. Some of these studies, called genome-wide association studies (GWAS), identify changes in the genetic code that impact on disease risk. These changes are often found in regions between genes, meaning that they are in regions that do not code for the building blocks in our body (proteins). How these changes can influence risk of disease can therefore be difficult to understand, and it may be that they affect how much of a particular protein is made, where the gene coding for that protein is nearby, but often this cannot be mathematically discerned from the data. Several studies had shown that DNA variations that occurred close to the *LYPLAL1* gene were associated with metabolic disease. As little was known about how this gene worked, we studied mice without any *Lyplal1* to see how this affected them. The mice were normal in all the metabolic tests we performed, which indicates that *LYPLAL1* is not important in controlling metabolism, and that this identified change may be acting through a different gene.

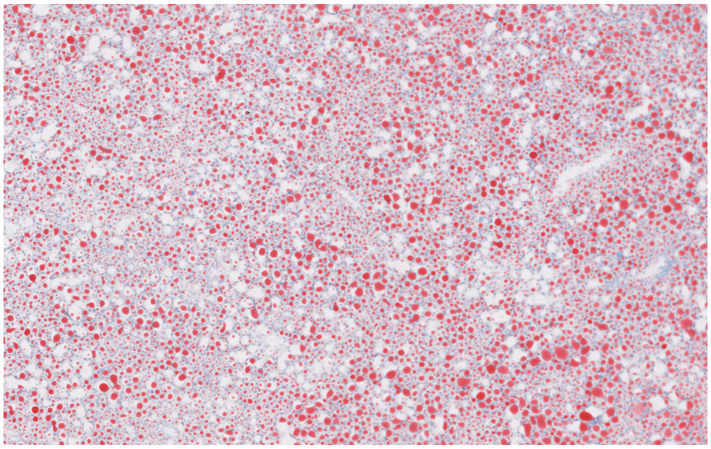

Fatty liver in a mouse fed a high-fat diet.

### What are the potential implications of these results for your field of research?

These results suggest that a different gene other than *LYLAL1* may be responsible for the signals identified by GWAS, which has up to now been attributed to *LYPLAL1*. The results also highlight the caution that must be taken when interpreting GWAS results. This has also been well documented in the case of the identification of the causal gene around the *FTO* locus.

### What are the main advantages and drawbacks of the model system you have used as it relates to the disease you are investigating?

Mice are an excellent model system for investigating metabolic disease. The increase in the use of human iPSCs has been very valuable to research; however, these cannot recreate the complexity of multicellular organism. Studies in mice allow us to investigate the effect on a living organism, which is particularly relevant for metabolic disease where phenotypes are affected by multiple organs, and where parameters such as food intake and exercise are important. Although it was not used in our study, the emergence of the CRISPR technology also allows much more rapid generation of mouse mutant models, where speed was previously a major drawback of this system.

### What has surprised you the most while conducting your research?

Given the number of papers linking LYPLAL1 to metabolic disease phenotypes, it was surprising to see no phenotype. However, as discussed above and in the paper, GWAS loci normally map to a non-coding region, and these are then suggested to be affecting the phenotype via the nearest gene, which is not always going to be

Rachel Watson's contact details: Wellcome Trust Sanger Institute, Wellcome Genome Campus, Hinxton, Cambridge CB10 1SA, UK.

E-mail: rw6@sanger.ac.uk

Disease Models & Mechanisms (2017): doi:10.1242/dmm.031864: Supplementary information

correct. This indicates the importance of functional follow-up of GWAS loci to confirm the effector gene.

**"…negative results are extremely important, as they can challenge widely held assumptions."**

### Describe what you think is the most significant challenge impacting your research at this time and how will this be addressed over the next 10 years?

Our paper demonstrates the absence of a phenotype in *Lyplal1* knockout mice. A major challenge affecting model organism research currently is the reluctance of many journals to publish such 'negative' results, and of researchers themselves to present them at conferences and to invest the time and effort to complete such studies in a rigorous manner once it appears likely that no phenotype is going to be discovered. However, as discussed in the paper, these negative results are extremely important, as they can challenge widely held assumptions, such as the assumption that *LYPLAL1* was the gene through which the GWAS loci were acting. In addition to the scientific value of negative results, it also has the consequence of causing a large waste in resources, as multiple different researchers generate similar models which never reach publication.

#### Reference

**Watson, R. A., Gates, A. S., Wynn, E. H., Calvert, F. E., Girousse, A., Lelliott, C. J. and Barroso, I.** (2017). *Lyplal1* is dispensable for normal fat deposition in mice. *Dis. Model. Mech.* **10**, doi:10.1242/dmm.031864.
